# Supplementary material for: Corner Flows Induced by Surfactant-Producing Bacteria Bacillus subtilis and Pseudomonas fluorescens
Source: Microbiol Spectr. 2022 Oct 10;10(5):e03233-22. doi: 10.1128/spectrum.03233-22 (PMC9603562; doi:10.1128/spectrum.03233-22)
Supplement: Supplemental file 5 — Fig. S1 to S3; captions to Movies S1 to S4. Download spectrum.03233-22-s0001.pdf, PDF file, 0.07 MB [file spectrum.03233-22-s0001.pdf]

Supplementary File

Corner flows induced by surfactant-producing bacteria *Bacillus subtilis* and *Pseudomonas fluorescens*

Yuan Li,[a,b] Joseph E. Sanfilippo,[c] Daniel Kearns,[d] Judy Q. Yang[a,b]#

#Address correspondence to Judy Yang, judyyang@umn.edu.

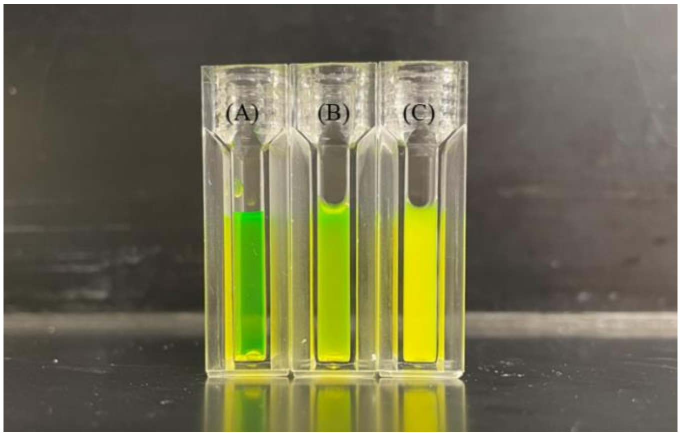

Fig. S1. The color change of bacteria solution under different bacterial densities. (A) Pure M9 culture with 0.005% (w/v) fluorescein sodium salt. (B) and (C) are *B. subtilis* culture at OD_{600} = 0.65 and OD_{600} = 1.8, respectively.

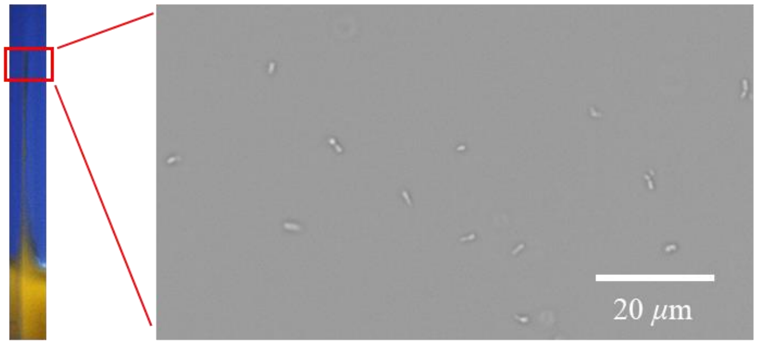

Fig. S2. (A)The left part is the image of *B. subtilis* (WT) culture at 30° corner in the chamber at t = 24 h. The right part is the confocal image of bacteria sampled from the tip of the corner flow at 30° corner. This proves that *B. subtilis* (WT) indeed migrated with the corner flow. (B) We use a mutant of *B. subtilis* which is lacking motility to repeat this experiment. The movement of this strain suggests corner flows are not due to bacterial motility. Note that the sampled bacterial solution was diluted with M9 solution by about 50 times to take the confocal photo.

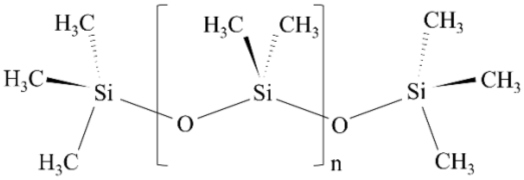

Fig. S3. Chemical structure of polydimethylsiloxane (PDMS).

Movie S1. The corner flow induced by *P. fluorescens* (WT), as shown in Fig. 3(A) of the main text. The duration of the video was 24 h.

Movie S2. The corner flow induced by *B. subtilis* (WT), as shown in Fig. 3(B) of the main text. The duration of the video was 24 h.

Movie S3. No corner flow was observed in the experiment with a surfactin-deficient mutant of *B. subtilis*, as shown in Fig. 3(C) of the main text. The duration of the video was 24 h.

Movie S4. The corner flow induced by *B. subtilis* (WT) in the chamber with three sharp corners of 30°, 50°, 60°, as shown in Fig.6 of the main text. The duration of the video was 24 h.
